# Unilateral Absence of the Latissimus Dorsi: A Report of a Rare Case

**DOI:** 10.7759/cureus.40346

**Published:** 2023-06-13

**Authors:** Hüseyin Erdem, Yigit Cevik, Nazire Kilic Safak, Ahmet Kursad Acikgoz, Gamze Taskin Senol

**Affiliations:** 1 Anatomy, Cukurova University Faculty of Medicine, Adana, TUR; 2 Anatomy, Abant Izzet Baysal University Hospital, Bolu, TUR

**Keywords:** cadaver, muscle absence, latissimus dorsi muscle, latissimus dorsi flap, latissimus dorsi

## Abstract

The latissimus dorsi is the largest muscle in the human body, located in the lower posterior thorax, and it contributes to motion in the upper extremity and provides assistance in respiration. This case report describes a rare occurrence of a unilateral absence of the latissimus dorsi accompanied by contralateral scoliosis in a 73-year-old female cadaver. The absence of the muscle along with the absence of the thoracodorsal nerve and branches of the subscapular and axillary arteries was identified during a standard dissection course. The implications of this rare case extend to an array of surgical interventions, including head, neck, breast, and torso reconstructive applications. It is important to investigate potential anomalies of the latissimus dorsi, while planning or performing free flap transfers. The report also highlights the importance of understanding this variation for educational and research purposes.

## Introduction

Muscular absences are a rare phenomenon, particularly in the pectoralis major and minor with an incidence of 0.061% in Japan and 0.013% in Europe [1]. Poland syndrome, which includes the congenital absence of the pectoralis major and/or pectoralis minor, has been relatively well studied, with recent approaches focusing on the surgical reconstruction of this anomaly [2]. In addition, there have been reports of single-sided absence of sternomastoid [3] and trapezius muscles [1], alterations in the pectoralis minor insertion [4], and absence of the latissimus dorsi [5,6]. Despite its rarity, with an estimated incidence of 1 in 36,000 to 50,000 births [1], these congenital muscular absences have implications for certain surgical procedures [2,7,8].

The latissimus dorsi is a large, broad muscle located in the lower posterior thorax, which serves as a primary contributor to motion in the upper extremity and functioning respiratory accessory muscle. Its attachments include the posterior iliac crest, the lower six spinous processes of thoracic vertebrae, the lower three to four ribs, and the inferior angle of the scapula. In addition, the muscle interdigitates with the external oblique, and through the thoracolumbar fascia, it attaches to the lumbar and sacral spinous processes and the supraspinous ligament. Its flat tendon attaches on the floor of the intertubercular sulcus [9].

Besides its locomotor and postural functions, the latissimus dorsi has an importance in reconstructive surgery [10]. The utilization of a latissimus dorsi flap is a viable treatment modality for repairing extensive soft-tissue defects of the head, neck, and torso [11,12]. This technique is highly advantageous due to its pliability and versatility, providing an optimal solution for a variety of reconstructive surgery procedures [10].

The authors have documented a rare and noteworthy case of a cadaver with a unilateral absence of the latissimus dorsi, which is similar to two cases previously reported in the literature [5,6]. This observation highlights the potential for anatomical variations and underscores the importance of thorough examination during anatomical dissections.

## Case presentation

This report describes a case of a unilateral absence of the latissimus dorsi muscle in a 73 year-old female cadaver. The cadaver was donated for educational and research purposes. The cause of death was reported as multiple-organ failure. There were no signs of trauma or post-surgical procedures.

**Figure 1:**
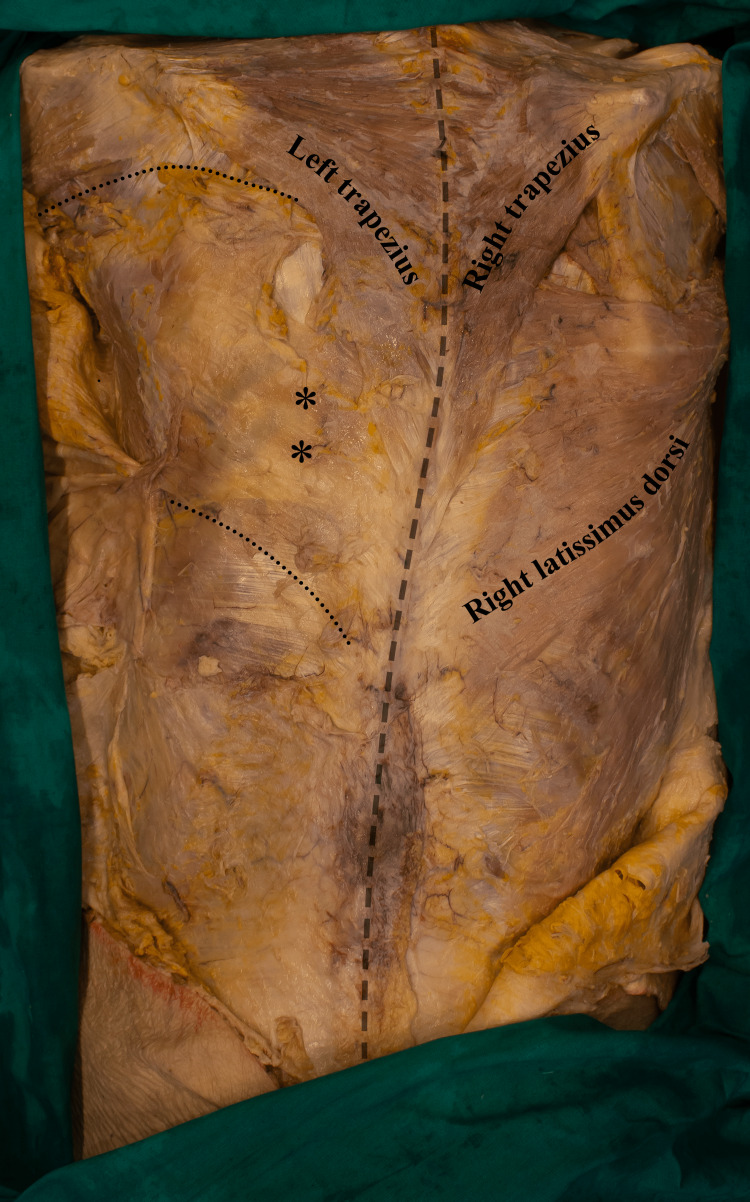
Superficial dissection of the torso (dorsal view). The dotted line represents the borders of the thin and fragile fascia extending along the T6 and T12. The transparent dashed line provides clarity on the course of the vertebral column, highlighting the dramatic scoliotic curvature toward the right side. *: small muscle fibers

During a standard dissection course, the left latissimus dorsi was not identified. However, small muscle fibers extending from the lower thoracic segments were visible (Figure 1). In addition, a very thin and fragile fascia extending through the T6 and T12 was determined (Figure 1). This fascia was not observed in the lumbar, sacral, and iliac segments. The left thoracodorsal nerve was absent, and the branches of the subscapular and axillary arteries supplying the muscle were not identified either. The vertebral column exhibited a concavity toward the right side, which was considered an evidence of scoliosis.

The latissimus dorsi on the contralateral side (right) demonstrated no discernible anatomical variations in its attachments or muscle fiber alignment (Figure 1).

The authors hereby confirm that every effort was made to comply with all local and international ethical guidelines and laws concerning the use of human cadaveric donors in anatomical research. Cukurova University Faculty of Medicine Non-Interventional Clinical Research Institutional Ethics Committee, Adana, Turkey, issued approval (protocol no.: 4.10.2023/131-14).

## Discussion

To the best of our knowledge, there have been no reported cases of the complete absence or extreme atrophy of the latissimus dorsi in a cadaver. Reports have documented this phenomenon by physical examinations, imaging modalities, and limited dissections in surgery [5,6].

The absence of the latissimus dorsi is considered a variation of Poland syndrome. The etiology of Poland syndrome is not fully understood, but it is thought to be caused by a vascular injury during the development of the embryo [13]. Other theories suggest it could be caused by a genetic mutation [13], an autoimmune response [14], or a teratogenic insult [15]. This syndrome has been associated with a variety of pathologies on the ipsilateral torso and upper extremity, including agenesis of the anterior ribs, malformation of the sternum, and absence of the serratus anterior, external abdominal oblique muscles, and the latissimus dorsi [5]. However, no variation or anomaly compatible with Poland syndrome was observed in this case except the absence of the latissimus dorsi and scoliosis.

There are only a handful of reports documenting such defects [5,6]. David and Winter reported the absence of the latissimus dorsi in a case of familial Poland syndrome involving two male members spanning two generations [5]. Moreover, Izadpanah et al. reported a case of latissimus dorsi absence observed during an ablative surgical procedure. Because it is such a rare condition, Izadpanah et al. did not consider the patient’s computed tomography (CT) images during preoperative evaluations to differentiate possible deficits in the surrounding musculature [6]. However, such anomalies should be elucidated to avoid any intra- or postoperative complications in free latissimus dorsi flap transfers.

The latissimus flap is an effective method of reconstructing extensive defects of the head, neck, and chest, especially when a broad soft-tissue coverage is required [10]. The pedicled latissimus dorsi transfer has been routinely employed in post-mastectomy breast reconstruction and scalp defect reparations [16,17]. Furthermore, the free functional muscle transfer of the latissimus dorsi has been utilized to facilitate facial reanimation and neophallus creation [8,18]. Nonetheless, in this case, the muscle was entirely absent with its attachments and neurovascular components. Preoperative imaging and detailed anatomical examination of the donor site could be crucial to identify potential variations in the muscle.

The role of the latissimus dorsi in postural control is well established, and disruptions in its symmetry have implications for the development of structural malformations, such as scoliosis [19,20]. In the current case, the absence of the latissimus dorsi on the left side may have led to a mechanical imbalance, resulting in the lateral deviation of the vertebral column toward the contralateral intact side (right side). This, in turn, evidently, contributed to the formation of a characteristic scoliotic curvature (Figure 1).

## Conclusions

This case report is noteworthy due to the rarity of the condition and to the fact that the cadaver had no history of trauma or surgical procedures. Despite its rarity, the absence of the latissimus dorsi is of significance when planning or performing free flap applications. Moreover, to provide a comprehensive understanding of the etiology of thoracolumbar scoliosis, a thorough assessment and clarification of potential anomalies or deficiencies in the adjacent musculature, such as the absence of the latissimus dorsi, could be crucial in planning or performing treatment strategies. It is also essential to accurately document such anomalies or variations encountered during anatomical dissections for educational and research purposes.
